# Methods for the optimal preservation of blow fly intra-puparial forms for morphological analysis in forensic casework

**DOI:** 10.1007/s00414-024-03172-9

**Published:** 2024-02-07

**Authors:** Jaime López-García, Mario A. Piña, Brett Clark, Martin J. R. Hall, Daniel Martín-Vega

**Affiliations:** 1 Universidad de Alcalá, Alcalá de Henares, Spain; 2 Universidad Nacional Autónoma de México, Ciudad de Mexico, Mexico; 3 Natural History Museum, London, UK

**Keywords:** Forensic entomology, Forensic science, minPMI, Puparium, Sampling protocol, Storage

## Abstract

Accurate minimum post-mortem interval (minPMI) estimations often rely on a precise age determination of insect developmental stages, which is significantly influenced by environmental temperature. An optimal preservation of the entomological samples collected at crime scenes is pivotal for a reliable aging of immature insect samples. For blow flies (Diptera: Calliphoridae), the most widely used insect indicators in forensic investigations, an appropriate preservation of tissues is particularly important in the case of puparial samples because aging methods for intra-puparial forms usually depend on morphological analyses; however, although informative soft tissues and structures could be discoloured and/or distorted if they are not properly fixed, there is a lack of studies to assess different methods for the optimal preservation of intra-puparial forms collected in forensic investigations. The present study compares three preservation methods for intra-puparial forms of the blow fly *Calliphora vicina* Robineau-Desvoidy, 1830: (i) direct immersion into 80% ethanol, (ii) puncturing of the puparium and hot water killing (HWK) prior to preservation in 80% ethanol, and (iii) HWK without puncturing before preservation in 80% ethanol. External and internal morphological analyses of intra-puparial forms of different ages were conducted to assess the quality of preservation. The results indicate that direct immersion in ethanol led to poor preservation, affecting both external and internal tissues. Both methods with HWK resulted in a better preservation, but puncturing resulted, in some cases, in physical damage of the specimens. HWK without puncturing emerged as the optimal preservation method, consistently yielding high preservation scores for both external and internal morphological analyses. These findings have practical implications for forensic practitioners and emphasise the need for updating some published guidelines and protocols in forensic entomology.

## Introduction

Blow flies (Diptera: Calliphoridae) are considered ideal forensic indicators as they generally colonise cadavers shortly after death, often within hours or even minutes [1, 2]. Colonisation refers to the egg-laying on the cadaver by a gravid female blow fly, so the newly hatched larvae can feed and develop on the fresh dead tissues. As the larvae feed and grow, they pass through three larval instars [3], with fully grown third-instar larvae generally dispersing from the cadaver in order to find a safe place to pupariate and undergo metamorphosis into an adult fly [4]. Due to their poikilothermia, the duration and developmental rates of the different stages of the life cycle are greatly dependant on the environmental temperature [1, 3]. If reliable temperature records and reference developmental data are available, an accurate minimum post-mortem interval (minPMI) can be provided based on the age estimation of the oldest developmental stage collected at the forensic scene [1, 5, 6].

While reliable temperature and reference developmental data are crucial for providing accurate minPMI estimates [5–7], appropriate preservation of the entomological material collected at the forensic scene is equally important. Appropriate fixation and preservation of samples avoids the decomposition of insect tissues, which could result in morphological and size changes that might lead to erroneous age estimations and, consequently, to inaccurate minPMI estimations. For blow fly larvae, whose length is typically used as a measure of age [3], hot water killing (HWK) prior to storage in 80–96% ethanol has long been the recommended fixation and preservation method [8–10]. However, for eggs, the widely used protocol developed by the European Association for Forensic Entomology [9] recommends killing and preserving the samples by direct immersion into 70–95% ethanol, without previous fixation. On the other hand, that protocol does not explicitly recommend any preservation method for living puparial samples (or ‘pupae’), although storage in 70–95% ethanol is recommended for all types of insect specimens [9]. Direct placement into ethanol has proven to result in a marked tissue decomposition and decolouration in both blow fly embryos and intra-puparial forms, whereas HWK prior to storage in ethanol improves the quality of preservation [11, 12]. An appropriate preservation of tissues is particularly important in the case of puparial samples because (i) aging methods often rely on internal and/or external morphological analyses of the intra-puparial forms [4, 13–17], and (ii) during most part of the intra-puparial period, the insect inside the protective puparium undergoes drastic histolytic and histogenetic processes and lacks a sclerotised cuticle [17, 18]; hence, informative soft tissues and structures could be discoloured and/or distorted if they are not properly fixed. In fact, puparial samples collected at the crime scene can be useless for an internal morphological analysis if they have been directly killed by immersion into ethanol leading to extensive tissue decomposition (Hall and Martín-Vega, personal observation).

The intra-puparial period accounts for over 50% of the developmental duration of the blow fly life cycle [4], so it can be a crucial timespan for estimating a minPMI and reliable age estimations of appropriately preserved samples are mandatory. In spite of its relevance, only one study to date [11] has assessed different methods for the optimal preservation of blow fly intra-puparial forms for minPMI estimations. The authors of that study compared different fixation methods, including HWK fixation, direct placement into 70% ethanol and a series of chemical fixatives, concluding that HWK prior to preservation in 80% ethanol was the optimal method for preservation of intra-puparial forms [11]. It must be highlighted that the authors punctured all the puparia three times (once in each tagma: head, thorax and abdomen) prior to HWK and indicated that puncturing is essential in order to enable an adequate HWK fixation [11]. However, puncturing puparia collected at the death scene might be impractical for forensic practitioners and poses the risk of damaging the insect inside. In fact, the authors noted pronounced discolouration around the puncturing incisions in the intra-puparial forms [11]. Taking into account that two of the available methods for aging blow fly intra-puparial forms are (i) external morphological analyses (mainly based on colouration changes) after puparia dissection [16, 17]; and (ii) internal morphological analyses of soft tissues and organs, using either traditional or ‘virtual’ histology [4, 14, 15], proper preservation while avoiding any potential physical damage of the intra-puparial forms is crucial.

The present study assesses the quality of preservation of intra-puparial forms for both external and internal morphological analyses in forensic casework, comparing three methods: (i) placing the puparial samples directly into 80% ethanol, following EAFE guidelines for the preservation of insect samples [9]; (ii) puncturing three times each puparial sample prior to HWK fixation and preservation in 80% ethanol, following the recommendation by Brown et al. [11]; and (iii) HWK fixing the puparial samples prior to storage in 80% ethanol, without puncturing the puparium. The aim of this study is to provide guidelines and recommendations for the optimal preservation of puparial samples analysed in forensic casework.

## Material and methods

### Insect culture and sampling

A laboratory colony of the blow fly *Calliphora vicina* Robineau-Desvoidy, 1830 was established using adults trapped with pig liver baits at the Scientific and Technological Campus of the University of Alcalá (Madrid, Spain). This blow fly species is among the first colonisers of cadavers and, as such, commonly used as a forensic indicator and as a target species for forensic entomology studies (e.g., [3, 4, 12–17]).

In the laboratory colony, the adults of *C. vicina* were provided with water, sugar cubes and milk powder ad libitum, whereas fresh pork liver was used as an oviposition substrate for gravid females. Once they had oviposited, groups of 100–200 eggs were transferred to plastic containers (12 cm x 12 cm × 7.5 cm) containing fresh pig liver as a feeding substrate for larvae and a layer of approximately 3 cm of vermiculite as a dispersal and pupariation substrate for post-feeding larvae. The plastic containers were placed into an incubator at a constant temperature (25 °C ± 0.8 °C) and 12:12 (L:D) photoperiod and, once the post-feeding larvae started to wander, the plastic containers were checked approximately every 6 h to collect the white prepupae [18], which were placed into separate plastic containers (12 cm x 12 cm × 7.5 cm) for each collection time. Following previous studies [4, 18], collection times for the puparia were established using percentages of the total intra-puparial period (IPP), with 0% corresponding to pupariation and 100% to adult emergence, and unpublished developmental data for *C. vicina* (López-García et al., in preparation).

### External morphological analyses

For external morphological analyses of the intra-puparial forms [13, 17], 100 puparia were collected at random at each 20, 50, 70 and 90% IPP, thus covering representative stages of the blow fly intra-puparial development [13, 18]. For each IPP percentage, the collected puparia were randomly divided into the following 4 treatment groups:

- Treatment 1: 25 puparia were immediately dissected using a scalpel to extract the living intra-puparial forms, which were photographed under a Leica® EZ4 D stereo microscope and used as control specimens. Each specimen was photographed on dorsal, lateral (both sides) and ventral position.
- Treatment 2: 25 puparia were killed by direct immersion into 80% ethanol and stored within this preservative at 4 °C for two weeks. Then, the puparia were dissected and the intra-puparial forms were extracted and photographed as described above.
- Treatment 3: 25 puparia were punctured three times, once in each tagma [11], using an entomological pin of size 2, killed and fixed in near boiling water for ∼30 s and subsequently stored in 80% ethanol at 4 °C for two weeks. Then, the puparia were dissected and the intra-puparial forms were extracted and photographed as described above.
- Treatment 4: 25 puparia were killed and fixed in near boiling water for ∼30 s, without puncturing, and subsequently stored in 80% ethanol at 4 °C for two weeks. Then, the puparia were dissected and the intra-puparial forms were extracted and photographed as described above.

**Fig. 1:**
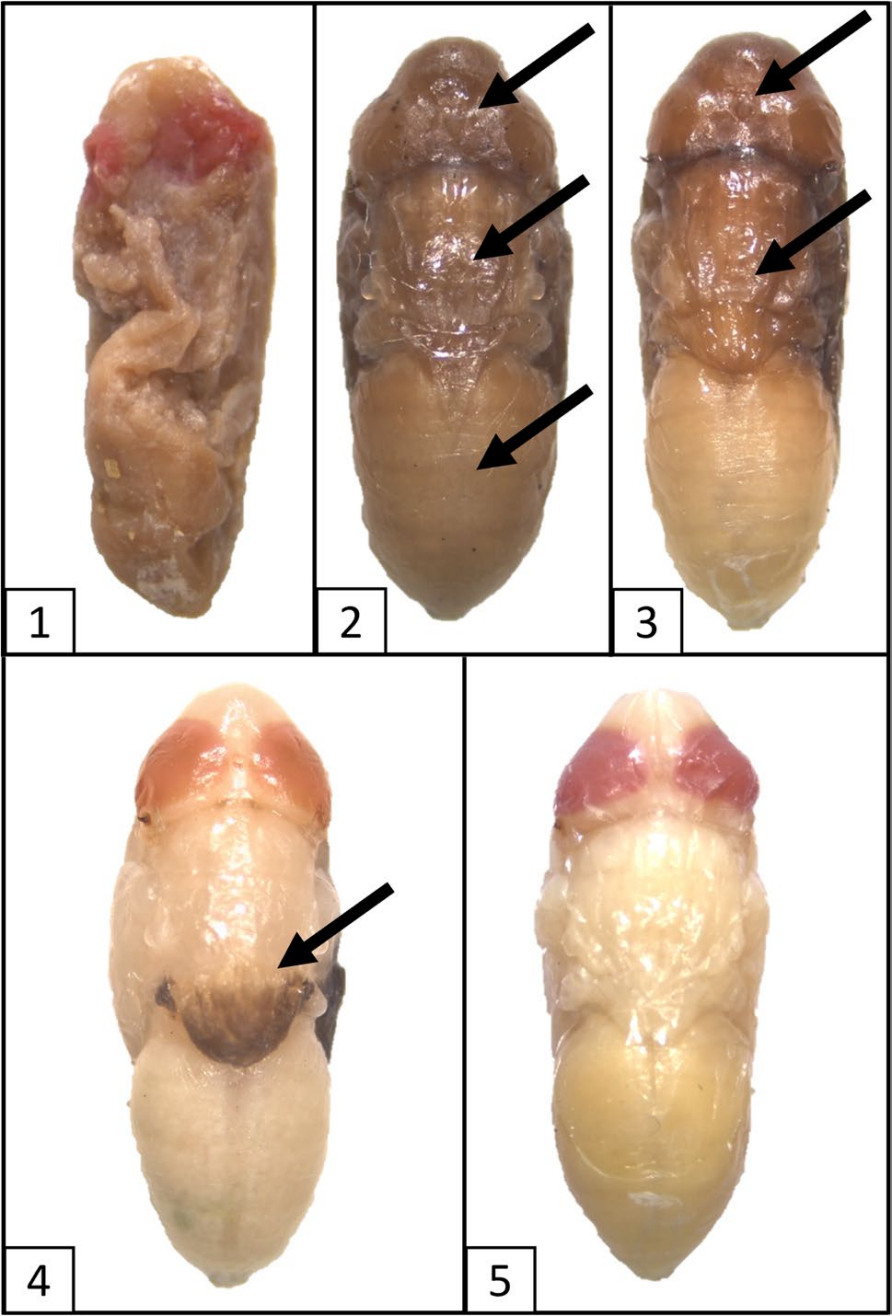
Sample of the different scores used to assess the preservation status of the intra-puparial forms of *Calliphora vicina*: 1) Completely collapsed. 2) Three damaged or obscured tagmata. 3) Two damaged or obscured tagmata. 4) One damaged or obscured tagma. 5) No damaged tagma, optimal condition. Damaged tagmata are marked by an arrow

For every treatment, the preservation status of each specimen was evaluated using a score of 1 to 5, assigned as follows: 1: completely collapsed; 2: three damaged or obscured tagmata; 3: two damaged or obscured tagmata; 4: one damaged or obscured tagma; 5: no damaged tagma, optimal condition (Fig. 1). In addition, for every treatment, each specimen was weighed on a Fisherbrand® Precision Series balance after being photographed. Prior to being weighed, the specimens from treatments 2–4 were gently dried on paper towel.

The entire procedure was replicated four times, using a different incubator each time to avoid potential bias. In total, 100 specimens (4 × 25 puparia) were analysed for each of the 4 considered IPP percentages (20, 50, 70 and 90%). Differences between treatments were analysed using a Kruskal–Wallis test and a Mann–Whitney pairwise test, with Bonferroni post-hoc corrections. Statistical analyses were performed using RStudio 1.1.463. Differences were considered to be significant at the *p* < 0.05 level.

### Internal morphological analyses

We compared the effect of treatments 2 and 4 on the preservation of the volume and appearance of internal tissues and organs using X-ray micro-computed tomography and following the methodology from previous studies, where treatment 4 had been used to fix and preserve blow fly puparia [4, 18]. Five puparia randomly collected at the same four percentages of the total IPP (25, 50, 75 and 90%) were stained by immersion in 0.5 M iodine in aqueous solution. Prior to immersion in the iodine solution, each puparium was punctured once in each tagma (head, thorax and abdomen) using an insect pin, to enhance the penetration of the staining solution [4, 18]. It is important to emphasize that the puncturing for staining was made after the direct placement and two-week storage in 80% ethanol (treatment 2) and after HWK fixation and two-week storage in 80% ethanol (treatment 4). The specimens were stained for 2 weeks and then washed and stored in 80% ethanol for 24 h before scanning. For scanning, each puparium was placed in a microcentrifuge tube containing 80% ethanol. Groups of 2–3 microcentrifuge tubes, each containing one puparium, were stacked inside a plastic pipette following the mounting set-up described in Martín-Vega et al. [19] and scanned in a Nikon Metrology HMX ST 225 system (current: 100 μA; voltage: 110 kV; exposure time: 500 ms). The resulting projections were reconstructed with a voxel size of 9.5 μm^3^ in CT-Pro 2.1 (Nikon Metrology, Tring, UK).

For each scanned puparium, the reconstructed slice stacks were rendered, reoriented and visualised in cross, horizontal and sagittal virtual sections using VG Studio Max 2.2 (Volume Graphics GmbH, Heidelberg, Germany). The reconstructed slice stacks were also loaded into Avizo 9.2 (Visualization Sciences Group, Bordeaux, France), where the pre-helicoidal region of the midgut was manually segmented for volume measurement. Segmentation refers to the process of labelling specific voxels (in this case, those belonging to the pre-helicoidal region of the midgut), enabling their analysis and visualisation separately from the other voxels. The pre-helicoidal region of the midgut was selected because it is one of the organs that undergoes the most drastic morphological and volumetric changes during the intra-puparial development; hence, it is a particularly informative structure for the aging of blow fly intra-puparial forms [4]. Volumetric measurements were performed using Avizo’s ‘Material Statistics’ module. Analyses of variance (ANOVA) were used to assess potential differences in the volume of the midgut within each percentage stage of the total IPP. The ANOVA were performed using Statgraphics Centurion (Statistical Graphics Corp. 1994–2000). Differences were considered to be significant at the *p* < 0.05 level.

## Results

### External morphological analyses

**Fig. 2:**
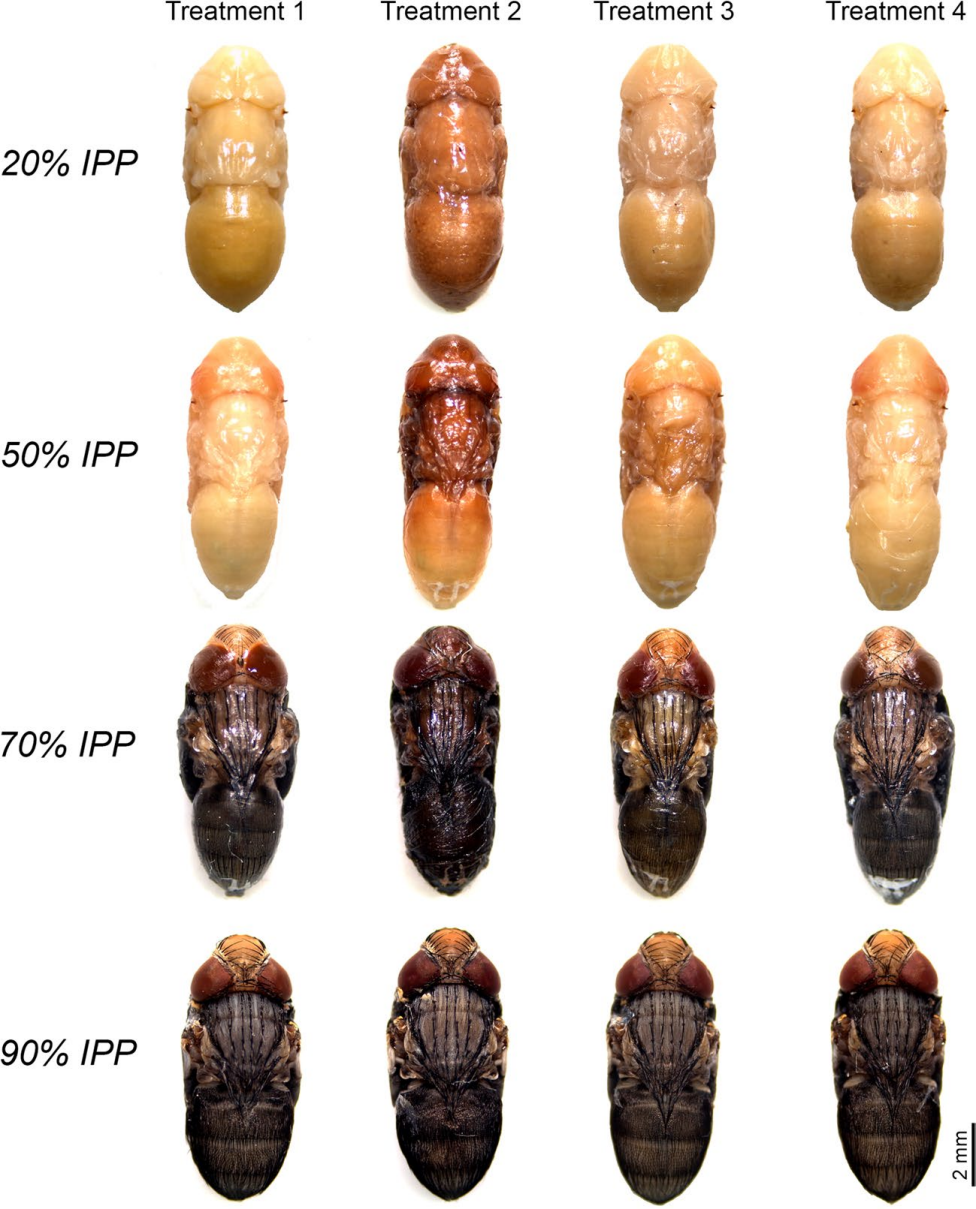
Sample of intra-puparial forms of *Calliphora vicina* collected at the different percentages of the total intra-puparial period (IPP) studied under different preservation methods: Treatment 1: living control samples; Treatment 2: samples killed and preserved into 80% ethanol; Treatment 3: puparia punctured prior to killing and fixation in near-boiling water and then preserved in 80% ethanol; Treatment 4: samples killed and fixed in near boiling water, without prior puncturing, and subsequently preserved in 80% ethanol

**Fig. 3:**
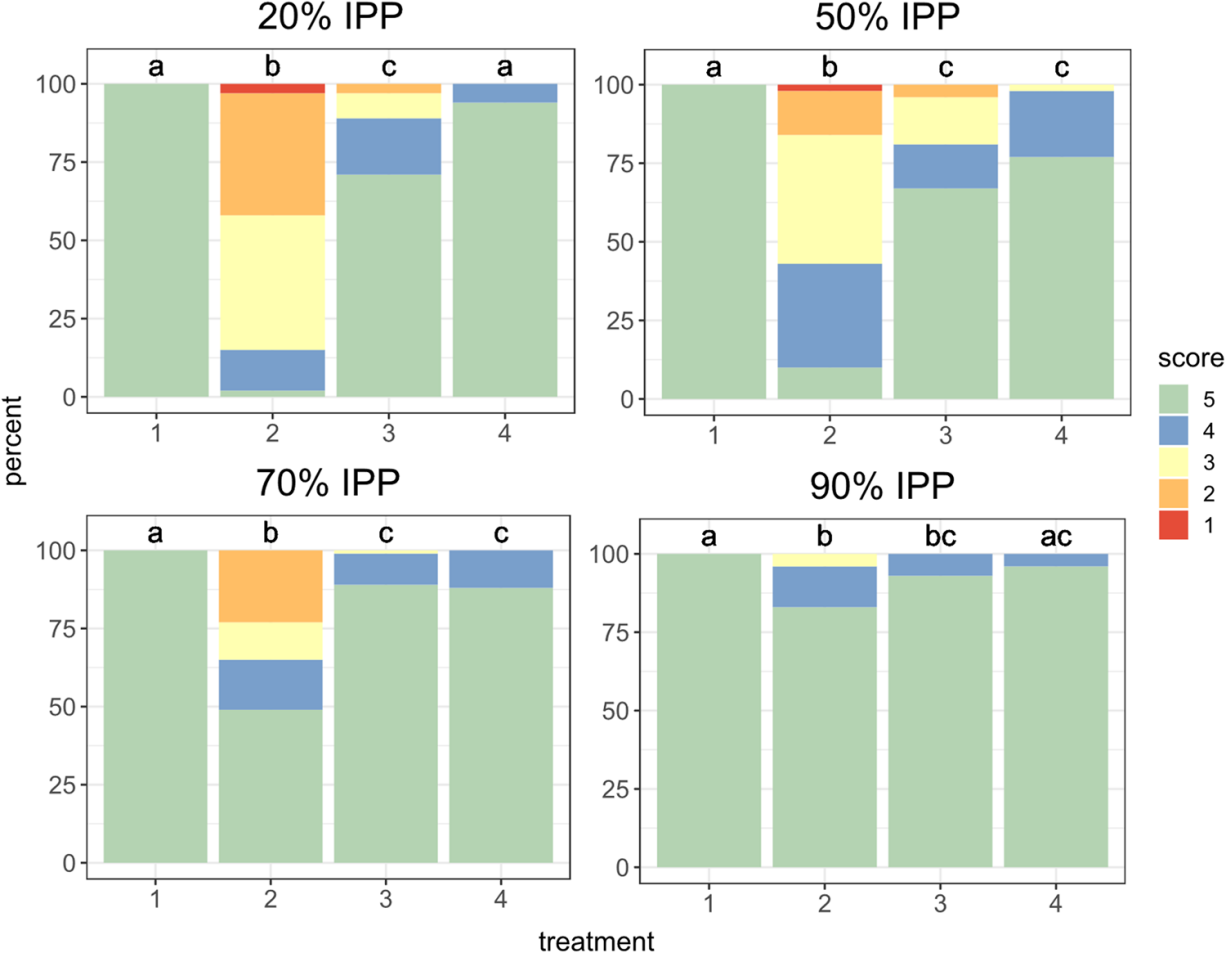
Preservation status of the intra-puparial forms of *Calliphora vicina* analysed by treatment for each percentage of the total intra-puparial period (IPP) studied. Treatment 1: living control samples. Treatment 2: samples killed and preserved into 80% ethanol. Treatment 3: puparia punctured prior to killing and fixation in near-boiling water and then preserved in 80% ethanol. Treatment 4: samples killed and fixed in near boiling water, without prior puncturing, and subsequently preserved in 80% ethanol. Significant differences between the different treatments are marked by different letters above each bar plot. Scores 1 to 5 used to assess the preservation status are illustrated in Fig. 1

Treatment 2, which involved killing the puparia by direct immersion in 80% ethanol and storage in the same solution, displayed the poorest preservation of coloration and turgor, particularly in specimens collected at 20% and 50% IPP, where discolouration and tissue decomposition was more evident (Fig. 2). Treatments 3 and 4, which involved killing and fixing the puparia in near boiling water for approximately 30 s, with or without puncturing, respectively, followed by preservation in 80% ethanol, resulted in higher preservation scores (Fig. 3). However, treatment 3 occasionally caused some physical damage due to puncturing of the puparium, observed in specimens collected at 20% and 50% IPP; i.e., those with softer tissues.

Kruskal–Wallis tests showed significant differences in preservation status between treatments in all the studied IPP percentages: 20% IPP (*N* = 400, *H* = 295.5, *p* < 0.05), 50% IPP (*N* = 400, *H* = 199.16, *p* < 0.05), 70% IPP (*N* = 400, *H* = 108.3, *p* < 0.05), and 90% IPP (*N* = 400, *H* = 24.57, *p* < 0.05).

Mann–Whitney pairwise comparisons revealed significant differences in preservation status between treatment 1 and both treatments 2 and 3 within all the studied IPP percentages (Table 1 and Fig. 3). Treatment 2, which overall resulted in the poorest preservation scores (Fig. 2), also showed significant differences with both treatments 3 and 4 (Fig. 3). Regarding the comparison between treatment 1 and 4, significant differences were only found within 50% IPP and 70% IPP. On the other hand, significant differences in the preservation status between treatments 3 and 4 were only observed at 20% IPP, but not within the other IPP percentages (Fig. 3). Although this suggests that both treatments 3 and 4 showed a similar preservation status, it must be emphasised that only treatment 4 did not show significant differences with treatment 1 in three of the four studied IPP percentages and it had overall higher preservation scores than treatment 3 (Fig. 3).

**Table 1:** Results from Mann–Whitney pairwise comparisons and corresponding *p*-values of the preservation status of the intra-puparial forms of *Calliphora vicina* between the different treatments for the studied percentages of the total intra-puparial period (IPP). Treatment 1: living control samples. Treatment 2: samples killed and preserved into 80% ethanol. Treatment 3: puparia punctured prior to killing and fixation in near-boiling water and then preserved in 80% ethanol. Treatment 4: samples killed and fixed in near boiling water, without prior puncturing, and subsequently preserved in 80% ethanol

Group	Compared treatments	U	p-value
*20% IPP*	1—2	100	< 0.05
	1—3	3550	< 0.05
	1—4	4700	0.08
	2—3	748.5	< 0.05
	2—4	145	< 0.05
	3—4	3817	< 0.05
*50% IPP*	1—2	500	< 0.05
	1—3	3350	< 0.05
	1—4	3850	< 0.05
	2—3	2022.5	< 0.05
	2—4	1068.5	< 0.05
	3—4	4310.5	0.2
*70% IPP*	1—2	2450	< 0.05
	1—3	4450	< 0.05
	1—4	4400	< 0.05
	2—3	2821.5	< 0.05
	2—4	2840	< 0.05
	3—4	4956	1
*90% IPP*	1—2	4150	< 0.05
	1—3	4650	< 0.05
	1—4	4800	0.27
	2—3	4486	0.16
	2—4	4342	< 0.05
	3—4	4850	1

**Fig. 4:**
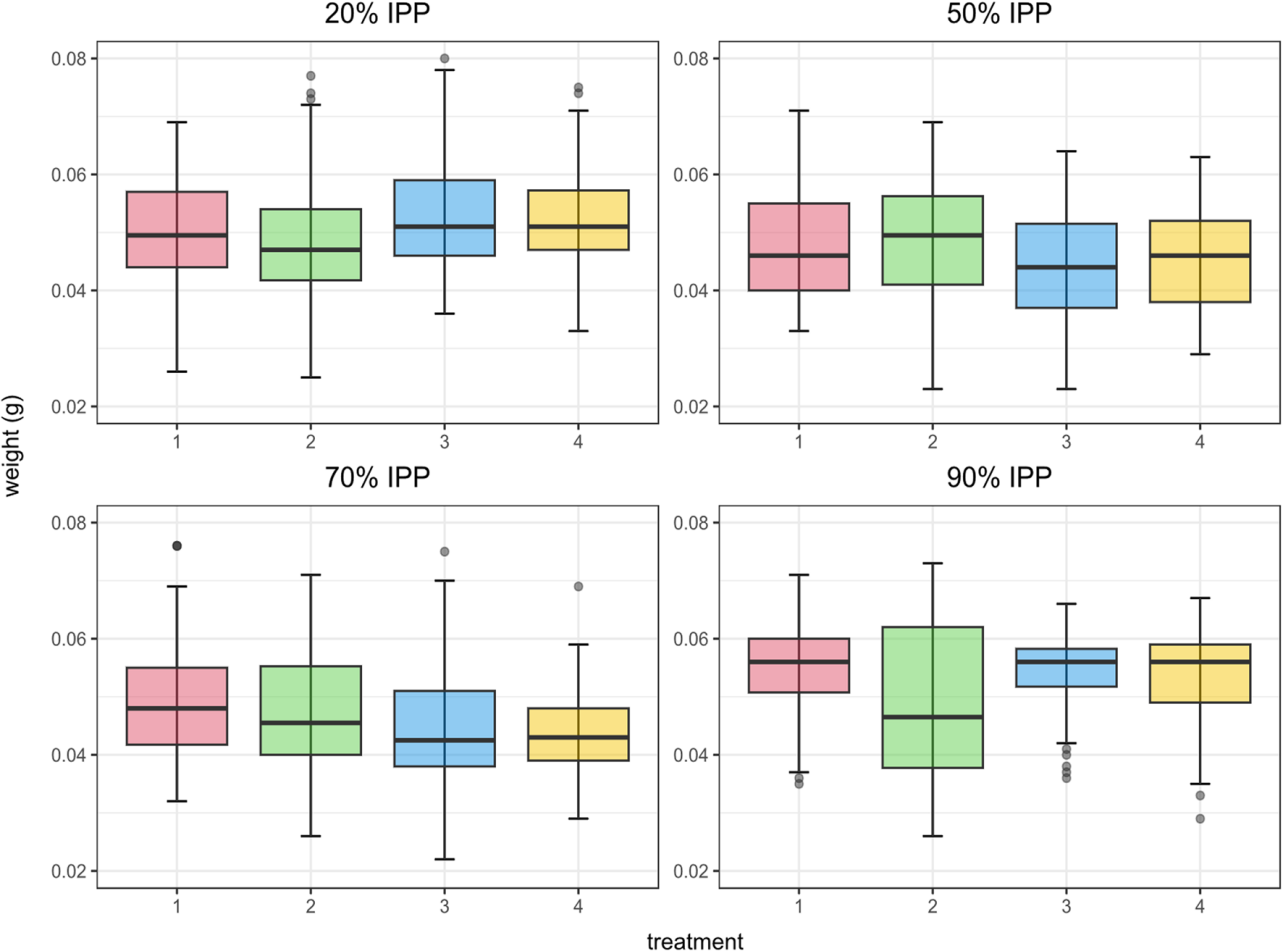
Boxplot representing the weight (g) of the intra-puparial forms of *Calliphora vicina* by treatment for each percentage of the total intra-puparial period (IPP) studied. The median is represented by the horizontal bar, the middle two quartiles are represented by the box, and the 99th percentile is represented by the whiskers. Treatment 1: living control samples. Treatment 2: samples killed and preserved into 80% ethanol. Treatment 3: puparia punctured prior to killing and fixation in near-boiling water and then preserved in 80% ethanol. Treatment 4: samples killed and fixed in near boiling water, without prior puncturing, and subsequently preserved in 80% ethanol

Regarding the weight of the intra-puparial forms, significant differences were found at 20% IPP between treatment 2 and treatments 3 and 4 (*U* = 3778, *p* < 0.05, *U* = 3829.5, *p* < 0.05, respectively), at 50% IPP between treatment 2 and treatment 3 (*U* = 3735, *p* < 0.05), and at 70% IPP between treatment 1 and treatments 3 and 4 (*U* = 3349, *p* < 0.05, *U* = 3268, *p* < 0.05, respectively). No significant differences were found at 90% IPP (Fig. 4).

### Internal morphological analyses

**Fig. 5:**
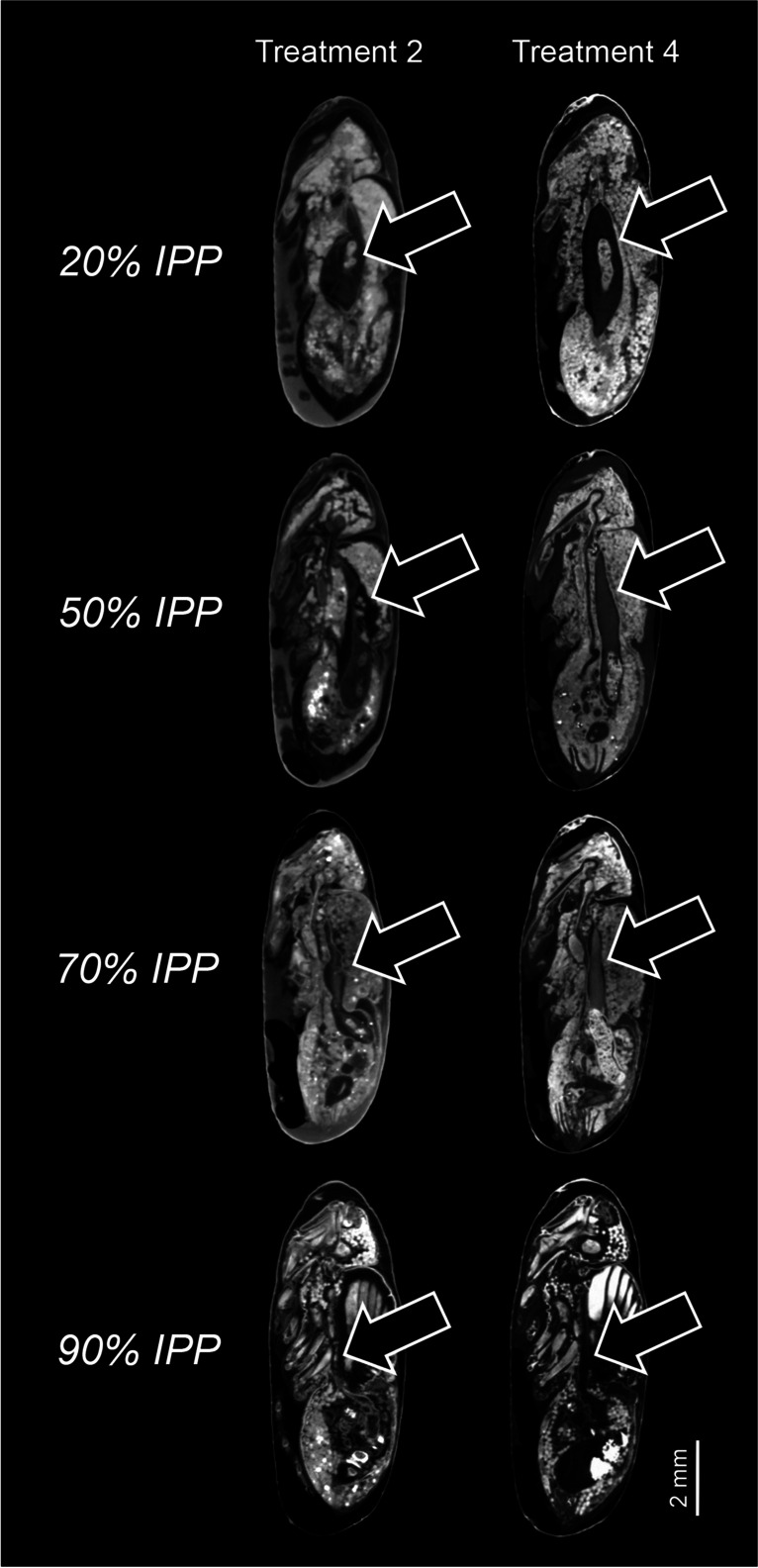
Micro-CT based virtual medial sagittal sections of *Calliphora vicina* puparia at each studied percentage of the total intra-puparial period (IPP), comparing treatment 2 (puparia directly preserved in 80% ethanol) and treatment 4 (samples killed in hot water, without puncturing the puparium, prior to preservation in 80% ethanol). Arrow indicates the midgut (dark grey area) containing the yellow body (light grey area)

Treatment 2 (specimens killed by direct immersion into 80% ethanol and preserved in 80% ethanol) resulted in a poor preservation of the internal tissues compared to specimens from treatment 4 (specimens killed and fixed in near boiling water for ∼30 s, without prior puncturing of the puparium, and subsequently preserved in 80% ethanol) (Fig. 5). Direct placement in ethanol without previous HWK fixation (treatment 2) resulted in a marked decomposition of the internal soft tissues, including the pre-helicoidal region of the midgut, which was particularly disfigured in specimens collected at 20, 50 and 70% IPP (Fig. 5).

**Fig. 6:**
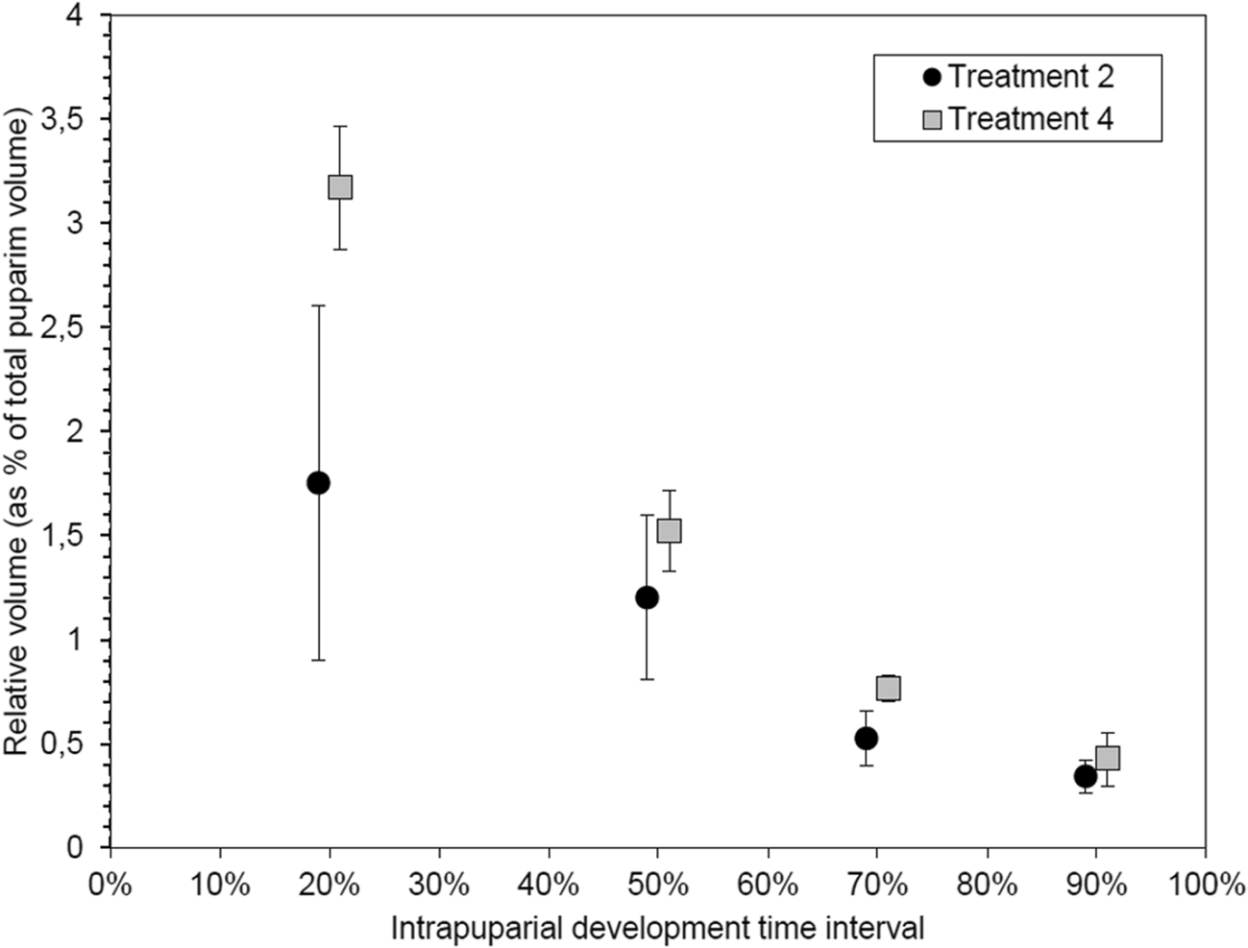
Average (± STD, n = 5) relative volume of the midgut at different percentages of the intra-puparial period of *Calliphora vicina* puparia directly preserved in 80% ethanol (treatment 2) and killed in hot water (without puncturing the puparium) prior to preservation in 80% ethanol (treatment 4)

Regarding the volume of the pre-helicoidal region of the midgut, specimens from treatment 2 collected at 20% IPP showed variable values, but, in every case, the volume was lower than those from treatment 4 (Fig. 6). Both the variation in volume among specimens from treatment 2 and the difference with the volume values from specimens from treatment 4 seemed to decrease as the developmental percentage of IPP increased (Fig. 6). Significant differences in the volume of the pre-helicoidal region of the midgut between treatments 2 and 4 were found at 20% IPP (F = 12.42, *p* < 0.05) and 70% IPP (F = 13.33, *p* < 0.05) IPP, but not at 50% IPP (F = 2.69, *p* > 0.05) and 90% IPP (F = 2.83, *p* > 0.05).

## Discussion

Studies aimed to test the suitability of different methods for the fixation, preservation and/or storage of immature blow fly samples in forensic casework have typically been focused on the larval stages (e.g., [8, 10, 20–24]), whereas egg and puparial samples have largely been neglected [11, 12]. An appropriate preservation of larval specimens is critical as their length is usually used as a measure of insect age in minPMI estimations [1]. Ethanol is widely used as a preservative for insect samples and other zoological material, as it is readily available and of low toxicity even at high concentrations [8]. Ethanol is indeed an excellent preservative of soft-bodied insect samples like the larvae of Diptera, but only if a proper fixation of the soft tissues has previously been performed. Direct killing of fly eggs and larvae in ethanol results in an extensive decomposition of tissues; hence, it should be avoided in forensic casework [8, 12]. The present study shows a similar effect of direct killing in ethanol on both the external and internal soft tissues of blow fly intra-puparial forms. The pupa and the developing adult of cyclorrhaphous Diptera are particularly soft and brittle, which explains the presence of a protective puparium as an almost exclusive structure of this group of flies [25]. Aging of intra-puparial forms usually relies on colour characters if an external morphological analysis is performed [13, 16, 17] or requires a proper preservation of internal organs and tissues if an either traditional or virtual histological analysis is performed [4, 14, 15]; therefore, direct placement of living puparial samples into ethanol should always be avoided as in the case of eggs and larvae [8, 25]. From a forensic perspective, discolouration and/or darkening resulting from direct preservation in ethanol, particularly within the first 50% IPP (Fig. 2), might be a potential source of error if the aging of the specimens is based on external colour characters, such as the degree of pigmentation of the eyes [17]. Similarly, direct preservation in ethanol could result in inaccurate estimates if the volume of an internal organ or structure, like the pre-helicoidal region of the midgut, is considered as a measure of age [4]. The unpredictability of the resulting preservation after direct placement into ethanol makes it difficult to estimate the range of its potential impact on minPMI estimations, particularly if a small number of puparial samples is available. In the worst-case scenario, completely collapsed intra-puparial specimens (Fig. 1.1) might prevent forensic practitioners from performing any morphological analysis at all.

HWK, which consists in killing insect specimens by immersion in nearly boiling water for a short period (∼30 s), has shown to be an effective and straightforward method for fixing blow fly larvae that enables both a reliable measuring of the body length and a species identification based on morphological characters [8, 9]. For blow fly eggs, HWK does not always result in a picture-perfect preservation of the embryo; nevertheless, it enables the observation of age-informative morphological landmarks [12]. Unlike some chemical fixatives widely used in histology [26], HWK provides a physical fixation as the heat results in the rapid coagulation of the internal tissues [8]. When applied to puparial samples, Brown et al. [11] stated that puncturing the puparium would be essential to facilitate an adequate penetration of the hot water because the thicker puparial cuticle may prevent the “heat transfer” and, therefore, the proper fixation of tissues. However, the present results show that puncturing is not needed, as it does not improve the HWK fixation of intra-puparial forms. HWK without prior puncturing results in an excellent fixation of tissues even in the Oestridae, a parasitic family of cyclorrhaphous Diptera characterised by a remarkably thick puparium [27]. Nevertheless, puncturing the puparium is required for an adequate penetration of the staining solution in micro-CT internal morphological analyses, but it must be highlighted that, in that case, puncturing must be performed after HWK fixation and on puparia already stored in ethanol [2, 18, 27]. Puncturing the puparium prior to HWK not only does not improve the fixation of tissues, but it poses the risk of damaging the insect inside and it is often a source of discolouration, as already noted by Brown et al. [11].

We observed significant differences in the weight of the samples between some treatments at certain percentages of the total IPP (Fig. 4); however, the observed differences were not consistent among the IPP percentages and did not show any particular effect. Future experiments should strengthen and refine the methodology to assess if weight can be used as a reliable age indicator and that water and/or ethanol potentially retained by sample tissues does not affect the results. Nonetheless, although a significant puparial weight loss during metamorphosis has been observed in *C. vicina* and other blow fly species of forensic relevance [14], aging methods of intra-puparial forms are mostly based on morphological analyses (e.g., [4, 13–17]); hence, optimal methods for the preservation of age-informative morphological markers should be prioritised rather than weight markers.

The results from the present study strongly support the use of HWK fixation prior to preservation in 80% ethanol for living puparial samples collected at the forensic scene for morphological analyses, with no need of puncturing the puparium prior to fixation. This method should greatly facilitate the work of those forensic practitioners who may not be properly trained in the handling of entomological samples, and it simplifies and unifies the guidelines for collecting entomological evidence [9]. In fact, a review and update of the published standards and guidelines for best practice in forensic entomology [9] would be desirable, in order to include further recommendations for the optimal morphological preservation of entomological samples (e.g., [10, 12, 22, 23, 28]), including specific preservation methods for molecular [29] and toxicological analyses [30].

## Data Availability

The data that support the findings of this study are available from the corresponding author upon reasonable request.
